# Prevalence of Symptoms ≤12 Months After Acute Illness, by COVID-19 Testing Status Among Adults — United States, December 2020–March 2023

**DOI:** 10.15585/mmwr.mm7232a2

**Published:** 2023-08-11

**Authors:** Juan Carlos C. Montoy, James Ford, Huihui Yu, Michael Gottlieb, Dana Morse, Michelle Santangelo, Kelli N. O’Laughlin, Kevin Schaeffer, Pamela Logan, Kristin Rising, Mandy J. Hill, Lauren E. Wisk, Wafah Salah, Ahamed H. Idris, Ryan M. Huebinger, Erica S. Spatz, Robert M. Rodriguez, Robin E. Klabbers, Kristyn Gatling, Ralph C. Wang, Joann G. Elmore, Samuel A. McDonald, Kari A. Stephens, Robert A. Weinstein, Arjun K. Venkatesh, Sharon Saydah, Innovative Support for Patients with SARS-CoV-2 Infections Registry (INSPIRE) Group, Innovative Support for Patients with SARS-CoV-2 Infections Registry (INSPIRE) Group, Zohaib Ahmed, Michael Choi, Antonia Derden, Michael Gottlieb, Diego Guzman, Minna Hassaballa, Ryan Jerger, Marshall Kaadan, Katherine Koo, Geoffrey Yang, Jocelyn Dorney, Jeremiah Kinsman, Shu-Xia Li, Zhenqiu Lin, Imtiaz Ebna Mannan, Senyte Pierce, Xavier Puente, Andrew Ulrich, Zimo Yang, Huihui Yu, Karen Adams, Jill Anderson, Gary Chang, Nikki Gentile, Rachel E. Geyer, Zenoura Maat, Kerry Malone, Graham Nichol, Jasmine Park, Luis Ruiz, Mary Schiffgens, Tracy Stober, Michael Willis, Zihan Zhang, Grace Amadio, Alex Charlton, David Cheng, Dylan Grau, Paavali Hannikainen, Efrat Kean, Morgan Kelly, Jessica Miao, Nicole Renzi, Hailey Shughart, Lindsey Shughart, Carly Shutty, Phillip Watts, Arun Kane, Peter Nikonowicz, Sarah Sapp, David Gallegos, Riley Martin, Chris Chandler, Megan Eguchi, Michelle L’Hommedieu, Raul Moreno, Kate Diaz Roldan, Mireya Arreguin, Virginia Chan, Cecilia Lara Chavez, Robin Kemball, Angela Wong, Melissa Briggs-Hagen, Aron J. Hall, Ian D. Plumb

**Affiliations:** ^1^Department of Emergency Medicine, University of California, San Francisco, San Francisco, California; ^2^Section of Cardiovascular Medicine, Department of Internal Medicine, Yale University, New Haven, Connecticut; ^3^Center for Outcomes Research and Evaluation, Yale New Haven Hospital, New Haven, Connecticut; ^4^Department of Emergency Medicine, Rush University Medical Center, Chicago, Illinois; ^5^Department of Emergency Medicine, University of Washington, Seattle, Washington; ^6^Department of Internal Medicine, Rush University Medical Center, Chicago, Illinois; ^7^Department of Emergency Medicine, University of Washington, Seattle, Washington; ^8^Department of Global Health, University of Washington, Seattle, Washington; ^9^Department of Emergency Medicine, Sidney Kimmel Medical College, Thomas Jefferson University, Philadelphia, Pennsylvania; ^10^National Center for Immunizations and Respiratory Diseases, CDC; ^11^Center for Connected Care, Sidney Kimmel Medical College, Thomas Jefferson University, Philadelphia, Pennsylvania; ^12^Department of Emergency Medicine, Sidney Kimmel Medical College, Thomas Jefferson University, Philadelphia, Pennsylvania; ^13^UTHealth Houston, Houston, Texas; ^14^University of California, Los Angeles, Los Angeles, California; ^15^Department of Emergency Medicine, Yale University, New Haven, Connecticut; ^16^University of Texas Southwestern Medical Center, Dallas, Texas; ^17^Division of General Internal Medicine and Health Services Research, David Geffen School of Medicine, University of California, Los Angeles, Los Angeles, California; ^18^Department of Health Policy and Management, Fielding School of Public Health, University of California, Los Angeles, Los Angeles, California; ^19^Department of Emergency Medicine, University of Texas Southwestern Medical Center, Dallas, Texas; ^20^Clinical Informatics Center, University of Texas Southwestern Medical Center, Dallas, Texas; ^21^Department of Family Medicine, University of Washington, Seattle, Washington; ^22^Department of Biomedical Informatics and Medical Education, University of Washington, Seattle, Washington; ^23^Department of Medicine, Division of Infectious Diseases, Rush University Medical Center, Chicago, Illinois; ^24^Department of Medicine, Division of Infectious Diseases, Cook County Hospital, Chicago, Illinois; ^25^Department of Internal Medicine, Yale University, New Haven, Connecticut.; Rush University; Rush University; Rush University; Rush University; Rush University; Rush University; Rush University; Rush University; Rush University; Rush University; Yale University; Yale University; Yale University; Yale University; Yale University; Yale University; Yale University; Yale University; Yale University; Yale University; University of Washington; University of Washington; University of Washington; University of Washington; University of Washington; University of Washington; University of Washington; University of Washington; University of Washington; University of Washington; University of Washington; University of Washington; University of Washington; University of Washington; Thomas Jefferson University; Thomas Jefferson University; Thomas Jefferson University; Thomas Jefferson University; Thomas Jefferson University; Thomas Jefferson University; Thomas Jefferson University; Thomas Jefferson University; Thomas Jefferson University; Thomas Jefferson University; Thomas Jefferson University; Thomas Jefferson University; Thomas Jefferson University; University of Texas Health Science Center at Houston; University of Texas Health Science Center at Houston; University of Texas Health Science Center at Houston; University of Texas Southwestern Medical Center; University of Texas Southwestern Medical Center; University of California, Los Angeles; University of California, Los Angeles; University of California, Los Angeles; University of California, Los Angeles; University of California, Los Angeles; University of California, San Francisco; University of California, San Francisco; University of California, San Francisco; University of California, San Francisco; University of California, San Francisco; CDC; CDC; CDC.

**Summary**

**What is already known about this topic?**

Post-COVID conditions, or long COVID, can persist for months or years after an acute COVID-19 illness and can include emergence of new symptoms or the occurrence of symptoms that come and go.

**What is added by this report?**

In a multicenter study of adults with a COVID-like illness, symptom prevalence decreased over time after the acute illness. Approximately 16% of adults with COVID-like symptoms reported persistent symptoms 12 months after a positive or negative SARS-CoV-2 test result. At 3, 6, 9, and 12 months after testing, some symptomatic persons had ongoing symptoms, and others had emerging symptoms not reported during the previous period.

**What are the implications for public health practice?**

Health care providers should be aware that symptoms can persist, emerge, reemerge, or resolve after COVID-like illness and are not unique to COVID-19 or to post-COVID conditions.

## Abstract

To further the understanding of post-COVID conditions, and provide a more nuanced description of symptom progression, resolution, emergence, and reemergence after SARS-CoV-2 infection or COVID-like illness, analysts examined data from the Innovative Support for Patients with SARS-CoV-2 Infections Registry (INSPIRE), a prospective multicenter cohort study. This report includes analysis of data on self-reported symptoms collected from 1,296 adults with COVID-like illness who were tested for SARS-CoV-2 using a Food and Drug Administration–approved polymerase chain reaction or antigen test at the time of enrollment and reported symptoms at 3-month intervals for 12 months. Prevalence of any symptom decreased substantially between baseline and the 3-month follow-up, from 98.4% to 48.2% for persons who received a positive SARS-CoV-2 test results (COVID test–positive participants) and from 88.2% to 36.6% for persons who received negative SARS-CoV-2 test results (COVID test–negative participants). Persistent symptoms decreased through 12 months; no difference between the groups was observed at 12 months (prevalence among COVID test–positive and COVID test–negative participants = 18.3% and 16.1%, respectively; p>0.05). Both groups reported symptoms that emerged or reemerged at 6, 9, and 12 months. Thus, these symptoms are not unique to COVID-19 or to post-COVID conditions. Awareness that symptoms might persist for up to 12 months, and that many symptoms might emerge or reemerge in the year after COVID-like illness, can assist health care providers in understanding the clinical signs and symptoms associated with post-COVID–like conditions.

## Introduction

Post-COVID conditions, or long COVID, comprise a range of symptoms that persist or develop ≥4 weeks after initial SARS-CoV-2 infection, and which are associated with substantial morbidity and reduced quality of life (*1*). Estimates of prevalence vary across settings, periods, and patient populations; and many studies lack comparison groups (*2*). Symptom trajectory over time using serial measurements has received little attention. Symptoms might either persist or emerge, and previous prevalence estimates typically include both persisting and emerging symptoms, without distinguishing between them (*1*,*2*).

## Methods

Innovative Support for Patients with SARS-CoV-2 Infections Registry (INSPIRE) is a prospective study including eight participating major health care systems,* designed to assess long-term symptoms and outcomes among persons with COVID-like illness at study enrollment who received a positive or negative SARS-CoV-2 test result^†^^,^^§^^,^^¶^ (COVID test–positive or COVID test–negative participants, respectively) (*2*). Participants could report subsequent SARS-CoV-2 positive test results at each follow-up survey. Participants who completed baseline and 3-, 6-, 9-, and 12-month follow-up surveys were included to facilitate distinguishing between emerging and ongoing symptoms. Outcomes included self-reported symptoms across eight symptom categories: 1) head, eyes, ears, nose, and throat (HEENT); 2) constitutional; 3) pulmonary; 4) musculoskeletal; 5) gastrointestinal; 6) cardiovascular; 7) cognitive difficulties; and 8) extreme fatigue (based on fatigue severity scales, which measure the occurrence and severity of eight symptoms associated with postinfectious syndrome; scores range from 10 to 80 and scores ≥25 correspond with previously established threshold for extreme fatigue).**^,^^††^ At each period, a participant was defined as having a persistent symptom if he or she had the symptom at that visit and all previous periods. Emerging symptoms were those present at a given time point but not present at the previous time point, including symptoms that resolved and reemerged after an absence.

Analyses included descriptions of the participants’ sociodemographic and clinical characteristics; statistical comparisons of the COVID test–positive and COVID test–negative groups were performed using Pearson’s chi-square tests. The prevalence of symptom persistence was defined as the proportion of participants with persistent symptoms at each time point; binomial 95% CIs were calculated for each outcome within each group and Pearson’s chi-square tests were used to test for differences in proportions. Symptom trajectories were reported as symptom prevalences at each time point, and the proportion of participants with emerging symptoms was also reported. All results are presented by symptom category, stratified by participants’ COVID test–positive and COVID test–negative status. Participants who reported a subsequent positive SARS-CoV-2 test result during the follow-up period were excluded from the analysis; as a sensitivity analysis, the same analysis was conducted for the entire cohort. Statistical analyses were performed using SAS software (version 9.4; SAS Institute). This study was approved by the institutional review boards at all eight institutions.^§§^

## Results

Among 6,075 enrolled participants, 3,726 (61%) completed the 12-month survey, 1,741 (47%) of whom completed all quarterly surveys through 12 months, including 1,288 COVID test–positive and 453 COVID test–negative participants, and are included in this study. Overall, 271 (21%) COVID test–positive participants reported a reinfection and 174 (38%) COVID test–negative participants reported a new infection during the 12-month follow-up period (p<0.01) and were excluded from the main analysis (Supplementary Figure 1, https://stacks.cdc.gov/view/cdc/131538). Approximately two thirds of participants identified as female (842; 67.4%) and 905 (72%) as non-Hispanic White (Table 1). Compared with the COVID test–negative group, a lower percentage of participants in the COVID test–positive group identified as female (65.2% versus 75.2%; p<0.01), and a higher percentage reported being married or living with a partner (60.3% versus 48.9%; p<0.01), and having been hospitalized for acute COVID-like illness (5.6% versus 0.4%; p<0.01). The prevalence of asthma was higher in the COVID test–negative group (18.3% versus 11.6%; p<0.01), as were the prevalences of kidney disease (2.5% versus 0.6%; p<0.01) and other unspecified conditions (20.1% versus 14.5%; p = 0.02).

**TABLE 1:** Self-reported characteristics of adults with acute COVID-like illness, by confirmed SARS-CoV-2 test result status* at time of enrollment — Innovative Support for Patients with SARS-CoV-2 Infections Registry study, United States, December 2020–March 2023

Characteristic^†^	No. (%)^§^			
	Overall (N = 1,296)	Positive test result (n = 1,017)	Negative test result (n = 279)	p-value
**Age group, yrs**				
18–34	**505 (39.3)**	388 (38.5)	117 (42.4)	0.31
35–49	**402 (31.3)**	327 (32.4)	75 (27.2)	
50–64	**266 (20.7)**	210 (20.8)	56 (20.3)	
≥65	**112 (8.7)**	84 (8.3)	28 (10.1)	
Missing	**11 (0.8)**	8 (0.8)	3 (1.1)	
**Gender**				
Female	**842 (67.4)**	642 (65.2)	200 (75.2)	<0.01
Male	**392 (31.4)**	329 (33.4)	63 (23.7)	
Transgender/Nonbinary/Other	**16 (1.3)**	13 (1.3)	3 (1.1)	
Missing	**46 (3.5)**	33 (3.2)	13 (4.7)	
**Hispanic or Latino^¶^**				
No	**1,105 (87.1)**	869 (87.2)	236 (86.4)	0.73
Yes	**164 (12.9)**	127 (12.8)	37 (13.6)	
Missing	**27 (2.1)**	21 (2.1)	6 (2.2)	
**Race^¶^**				
Asian	**149 (11.9)**	107 (10.9)	42 (15.6)	0.13
Black or African American	**96 (7.6)**	73 (7.4)	23 (8.5)	
White	**905 (72.1)**	724 (73.5)	181 (67.0)	
Other/Multiple	**105 (8.4)**	81 (8.2)	24 (8.9)	
Missing	**41 (3.2)**	32 (3.1)	9 (3.2)	
**Education**				
Less than high school diploma	**11 (0.9)**	9 (0.9)	2 (0.7)	0.11
High school graduate or GED certificate	**82 (6.5)**	65 (6.5)	17 (6.3)	
Some college but did not complete degree	**195 (15.4)**	143 (14.4)	52 (19.1)	
2-year college degree	**100 (7.9)**	75 (7.5)	25 (9.2)	
4-year college degree	**420 (33.1)**	348 (35.0)	72 (26.5)	
More than 4-year college degree	**459 (36.2)**	355 (35.7)	104 (38.2)	
Missing	**29 (2.2)**	22 (2.2)	7 (2.5)	
**Marital status**				
Never married	**416 (32.1)**	309 (30.4)	107 (38.5)	<0.01
Married/Living with a partner	**749 (57.8)**	613 (60.3)	136 (48.9)	
Divorced/Widowed/Separated	**130 (10.0)**	95 (9.3)	35 (12.6)	
Missing	**1 (0.1)**	0 (—)	1 (0.4)	
**Where COVID-19 testing was received**				
At-home testing kit	**75 (5.8)**	57 (5.6)	18 (6.5)	<0.01
Tent/Drive-up testing site	**726 (56.2)**	601 (59.4)	125 (44.8)	
Clinic including an urgent care clinic	**212 (16.4)**	161 (15.9)	51 (18.3)	
Hospital	**114 (8.8)**	82 (8.1)	32 (11.5)	
Emergency department	**69 (5.3)**	46 (4.5)	23 (8.2)	
Other	**95 (7.4)**	65 (6.4)	30 (10.8)	
Missing	**5 (0.4)**	5 (0.5)	0 (—)	
**Health insurance**				
Private and public	**52 (4.0)**	34 (3.3)	18 (6.5)	<0.01
Private only	**935 (72.1)**	749 (73.6)	186 (66.7)	
Public only	**264 (20.4)**	195 (19.2)	69 (24.7)	
None	**45 (3.5)**	39 (3.8)	6 (2.2)	
**Hospitalization**				
No	**1,218 (95.5)**	943 (94.4)	275 (99.6)	<0.01
Yes	**57 (4.5)**	56 (5.6)	1 (0.4)	
Missing	**21 (1.6)**	18 (1.8)	3 (1.1)	
**Preexisting medical condition**				
Asthma (moderate or severe)	**169 (13.0)**	118 (11.6)	51 (18.3)	<0.01
Hypertension or high blood pressure	**182 (14.0)**	137 (13.5)	45 (16.1)	0.26
Diabetes	**72 (5.6)**	50 (4.9)	22 (7.9)	0.06
Overweight or obesity	**352 (27.2)**	272 (26.7)	80 (28.7)	0.52
Emphysema or COPD	**12 (0.9)**	9 (0.9)	3 (1.1)	0.77
Heart conditions such as CAD, heart failure, or cardiomyopathies	**30 (2.3)**	19 (1.9)	11 (3.9)	0.04
Tobacco use (currently using any type of tobacco, including smokeless tobacco)	**61 (4.7)**	44 (4.3)	17 (6.1)	0.22
Kidney disease	**13 (1.0)**	6 (0.6)	7 (2.5)	<0.01
Liver disease	**15 (1.2)**	9 (0.9)	6 (2.2)	0.08
Other	**203 (15.7)**	147 (14.5)	56 (20.1)	0.02
Participants reporting emerging symptoms at 6–12 mos**				
Any symptom^††^	**11 (0.9)**	1 (0.1)	10 (3.7)	<0.01
HEENT	**30 (2.4)**	10 (1.0)	20 (7.5)	<0.01
Constitutional	**27 (2.1)**	9 (0.9)	18 (6.7)	<0.01
Pulmonary	**51 (4.1)**	28 (2.8)	23 (8.6)	<0.01
Musculoskeletal	**66 (5.3)**	42 (4.2)	24 (9.0)	<0.01
Gastrointestinal	**56 (4.5)**	34 (3.4)	22 (8.2)	<0.01
Cardiovascular	**60 (4.8)**	42 (4.2)	18 (6.7)	0.09
Cognitive difficulties	**107 (8.3)**	68 (6.7)	39 (14.0)	<0.01
Extreme fatigue	**90 (7.0)**	65 (6.5)	25 (9.1)	0.13

**Abbreviations:** CAD = coronary artery disease; COPD = chronic obstructive pulmonary disease; GED = general educational development; HEENT = head, ears, eyes, nose, and throat.

* Excluding participants who reported receiving a negative test result during follow-up.

**^†^** Data were recorded at time of enrollment. The preexisting conditions data were collected at 3 months follow-up, which resulted in the high level of missingness in these variables.

^§^ Calculation of percentage and p-values excluded cases with missing values.

^¶^ Persons of Hispanic or Latino (Hispanic) origin might be of any race but are categorized as Hispanic; all racial groups are non-Hispanic.

** Symptom categories were any symptom (one or more symptoms), HEENT (headache, runny nose, loss of smell, loss of taste, sore throat, and loss of hair), constitutional (tired, chills, feeling hot, fever, and shakes), pulmonary (cough, shortness of breath, and wheezing), musculoskeletal (aches and joint pains), gastrointestinal (diarrhea, nausea or vomiting, and abdominal pain), cardiovascular (chest pain and palpitations), cognitive difficulties (forgetfulness/memory problems, difficulty thinking, or difficulty concentrating), and extreme fatigue (fatigue severity score ≥25).

**^††^** Among participants who did not have any symptom at time of enrollment or 3 months after a COVID-like illness.

Symptom prevalence at baseline and persistence through 12 months varied according to symptom category (Table 2). A higher proportion of COVID test–positive participants reported symptoms in each category, except for extreme fatigue, at baseline compared with COVID test–negative participants. Symptom prevalence declined over time within each symptom category: 18.3% of COVID test–positive participants and 16.1% of COVID test–negative participants reported persistent symptoms of any type through 12 months. Symptom persistence through 12 months for a given symptom category ranged from 0.3% (gastrointestinal symptoms) to 5.9% (HEENT symptoms) among COVID test–positive participants and from 1.1% (cardiovascular symptoms or pulmonary symptoms) to 6.8% (extreme fatigue) among COVID test–negative participants. Only the persistence of extreme fatigue was statistically significantly different at 12 months between COVID test–positive participants (3.5%) and COVID test–negative participants (6.8%).

**TABLE 2:** Self-reported symptom* prevalence at baseline and persistence^†^ through 12 months after a COVID-like illness among adults, by SARS-CoV-2 test status^§^ — Innovative Support for Patients with SARS-CoV-2 Infections Registry, United States, December 2020–March 2023

Symptoms	Test result	Prevalence, % (95% CI)				
		Baseline	3 mos	6 mos	9 mos	12 mos
Any symptom	Positive	98.4 (97.7–99.2)	48.2 (45.1–51.3)	31.2 (28.3–34.0)	24.4 (21.7–27.0)	18.3 (15.9–20.7)
	Negative	88.2 (84.4–92.0)	36.6 (30.9–42.2)	22.2 (17.3–27.1)	17.9 (13.4–22.4)	16.1 (11.8–20.4)
HEENT	Positive	93.2 (91.7–94.8)	30.6 (27.7–33.4)	15.2 (13.0–17.4)	9.2 (7.5–11.0)	5.9 (4.5–7.3)
	Negative	73.5 (68.3–78.7)	19.0 (14.4–23.6)	10.0 (6.5–13.6)	7.5 (4.4–10.6)	5.4 (2.7–8.0)
Constitutional	Positive	86.4 (84.3–88.5)	22.5 (20.0–25.1)	9.4 (7.6–11.2)	4.8 (3.5–6.1)	2.9 (1.8–3.9)
	Negative	62.7 (57.1–68.4)	17.6 (13.1–22.0)	8.2 (5.0–11.5)	5.0 (2.5–7.6)	2.9 (0.9–4.8)
Pulmonary	Positive	68.0 (65.2–70.9)	11.0 (9.1–12.9)	3.9 (2.7–5.1)	2.0 (1.1–2.8)	1.4 (0.7–2.1)
	Negative	44.1 (38.3–49.9)	7.2 (4.1–10.2)	2.2 (0.4–3.9)	1.4 (0–2.8)	1.1 (0–2.3)
Musculoskeletal	Positive	60.6 (57.6–63.6)	13.3 (11.2–15.4)	6.1 (4.6–7.6)	3.6 (2.5–4.8)	2.0 (1.1–2.8)
	Negative	40.9 (35.1–46.6)	8.6 (5.3–11.9)	3.2 (1.2–5.3)	2.5 (0.7–4.3)	2.2 (0.4–3.9)
Gastrointestinal	Positive	34.0 (31.1–36.9)	4.8 (3.5–6.1)	1.7 (0.9–2.5)	0.7 (0.2–1.2)	0.3 (0–0.6)
	Negative	26.5 (21.3–31.7)	5.7 (3.0–8.5)	1.8 (0.2–3.3)	1.4 (0–2.8)	1.1 (0–2.3)
Cardiovascular	Positive	25.3 (22.6–27.9)	4.7 (3.4–6.0)	1.5 (0.7–2.2)	1.0 (0.4–1.6)	0.7 (0.2–1.2)
	Negative	17.2 (12.8–21.6)	3.6 (1.4–5.8)	1.4 (0–2.8)	1.1 (0–2.3)	1.1 (0–2.3)
Cognitive difficulties	Positive	25.0 (22.3–27.6)	9.2 (7.5–11.0)	6.4 (4.9–7.9)	4.5 (3.2–5.8)	3.8 (2.7–5.0)
	Negative	21.5 (16.7–26.3)	7.5 (4.4–10.6)	5.7 (3.0–8.5)	3.6 (1.4–5.8)	3.2 (1.2–5.3)
Extreme fatigue	Positive	21.1 (18.6–23.7)	8.1 (6.4–9.7)	6.0 (4.5–7.5)	4.4 (3.2–5.7)	3.5 (2.4–4.7)
	Negative	25.4 (20.3–30.6)	11.5 (7.7–15.2)	7.5 (4.4–10.6)	7.2 (4.1–10.2)	6.8 (3.9–9.8)

**Abbreviation:** HEENT = head, ears, eyes, nose, and throat.

* Symptom categories were any symptom (one or more symptoms), HEENT (headache, runny nose, loss of smell, loss of taste, sore throat, and loss of hair), constitutional (tired, chills, feeling hot, fever, and shakes), pulmonary (cough, shortness of breath, and wheezing), musculoskeletal (aches and joint pains), gastrointestinal (diarrhea, nausea or vomiting, and abdominal pain), cardiovascular (chest pain and palpitations), cognitive difficulties (forgetfulness/memory problems, difficulty thinking, or difficulty concentrating), and extreme fatigue (fatigue severity score ≥25). Percentage of participants reporting symptoms at each of the time points is presented for each symptom category, stratified by SARS-CoV-2 test result status at time of enrollment.

^†^ Persistent symptoms were defined as those present at time of enrollment and reported at each follow-up time point. Binomial 95% CIs were calculated for each outcome within each group. Pearson’s chi-square tests were used to test for differences in proportions at each time point.

^§^ Without evidence of new SARS-CoV-2 infection.

During the follow-up period, the symptom prevalences in each category except for extreme fatigue were similar at each time point for both COVID test–positive and COVID test–negative participants (Figure). Overall, no difference in symptom prevalence between COVID test–positive and COVID test–negative participant groups was observed across the four periods for the nine total symptom categories. Among COVID test–negative participants, prevalence of extreme fatigue was higher at 9 and 12 months compared to the COVID test–positive group. Approximately one half of participants in each group experienced any symptom at 12 months. Emerging symptoms were reported for every symptom category at each follow-up period for both groups. COVID test–negative participants reported higher prevalences of emerging symptoms at 6 and 12 months in each of the symptom categories, except severe fatigue (Table 1). When participants who reported a subsequent positive SARS-CoV-2 test result were included, the observed pattern was similar to that in the primary analysis, with more statistically significant differences in symptom prevalence during the follow-up period (Supplementary Figure 2, https://stacks.cdc.gov/view/cdc/131538) (Supplementary Figure 3, https://stacks.cdc.gov/view/cdc/131538).

**FIGURE:**
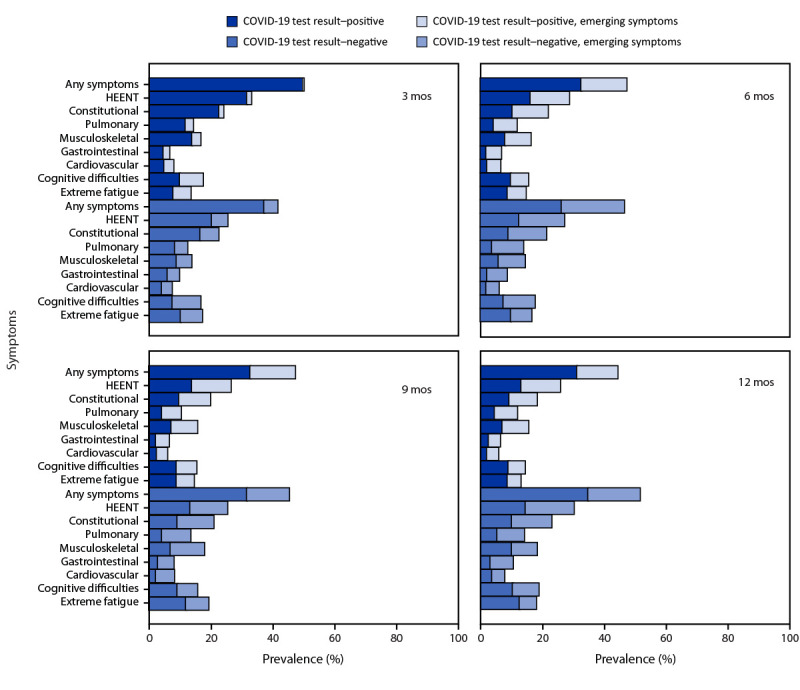
Self-reported prevalence of emerging and reemerging symptoms,*^,^^†^^,^^§^ by symptom category during 12 months^¶^ among adults with an acute COVID-like illness with no evidence of new or reinfection by SARS-CoV-2 test result status** — Innovative Support for Patients with SARS-CoV-2 Infections Registry, United States, December 2020–March 2023 **Abbreviation:** HEENT = head, ears, eyes, nose, and throat. * Symptom categories were any symptom (one or more symptoms), HEENT (headache, runny nose, loss of smell, loss of taste, sore throat, and loss of hair), constitutional (tired, chills, feeling hot, fever, and shakes), pulmonary (cough, shortness of breath, and wheezing), musculoskeletal (aches and joint pains), gastrointestinal (diarrhea, nausea or vomiting, and abdominal pain), cardiovascular (chest pain and palpitations), cognitive difficulties (forgetfulness/memory problems, difficulty thinking, or difficulty concentrating), and extreme fatigue (fatigue severity score ≥25). ^†^ Emerging symptoms were symptoms present at a given time point but not at the previous time point, including symptoms that resolved and reemerged after an absence. ^§^ https://www.cdc.gov/me-cfs/pdfs/wichita-data-access/symptom-inventory-doc.pdf ^¶^ Point prevalence at each time point is presented for the COVID test result–positive and COVID test result–negative groups for each symptom category. ** Without evidence of reinfection.

## Discussion

In this prospective, multicenter study of 1,296 persons with acute COVID-like illness, approximately 16% of participants reported persistent symptoms 12 months after their illness, irrespective of their SARS-CoV-2 test result status at baseline. A higher proportion of COVID test–positive than COVID test–negative participants reported symptoms in each symptom category at baseline. The prevalence of symptoms declined substantially in both groups from baseline to the 3-month follow-up assessment and continued to gradually decrease at the 6-, 9-, and 12-month follow-up assessments; persistence of any symptom prevalence at 12 months was not statistically significantly different between the COVID test–positive (18.3%) and COVID test–negative (16.1%) participant groups.

These findings expand the understanding of post-COVID conditions. Previous studies have reported symptom prevalence estimates across varied, nonstandardized periods or at a single point in time, resulting in challenges comparing studies and difficulty distinguishing among the presence of reported persistent symptoms at the time of COVID-19 diagnosis, those that resolved and then reemerged, and those that emerged after initial recovery (*3*–*9*). Few previous longitudinal studies have compared symptoms in COVID test–positive participants with those in persons with a COVID-like illness and who received negative SARS-CoV-2 test results. By conducting serial measurements of emerging and ongoing symptoms, this study was able to ascertain that participants who were symptomatic at a given time point included participants with ongoing symptoms as well as those with emerging symptoms (i.e., symptoms that were not present 3 months earlier). The inclusion of participants with COVID-like illness and negative test results guides discussions on characterizing symptoms associated with post-COVID conditions (*10*). This differentiation adds nuance and clarity to the natural history of post-COVID conditions and characterizes the fluctuating nature of symptoms over time and recognizes that these symptoms are not unique to COVID-19 or to post-COVID conditions. Many participants experienced new symptoms ≥6 months after the acute illness, suggesting that the prevalence of emerging symptoms in the months after acute COVID-like illness might be considerable. Cognitive difficulties and extreme fatigue were two common symptoms that emerged after 6 months and are often reported to occur with post-COVID conditions (*1*,*3*,*6*,*9*). Differentiating between symptoms that resolve and emerge over time helps to characterize post-COVID conditions and suggests that measurements at single time points underestimate or mischaracterize the true effects of disease.

### Limitations

The findings in this report are subject to at least four limitations. First, among the COVID test–negative group, no information on any other condition that might have caused the reported acute symptoms is available. Second, although the number of participants who subsequently reported a positive SARS-CoV-2 test result was higher in the COVID test–negative than in the COVID test–positive group, the rate of nonresponse to the question about having a subsequent SARS-CoV-2 test result was relatively higher in the COVID test–negative group. Testing was not systematically performed and participants with a subsequent SARS-CoV-2 infection might have not tested or might have received a false-negative test result. However, analysis including participants who reported subsequent positive test results did not differ substantially; thus, the results are not likely driven by subsequent SARS-CoV-2 infections. Infection with any other pathogen or the occurrence of other medical problems might have been experienced by persons in either group and could account for some reported symptoms. Third, the survey did not include all possible symptoms; therefore, other symptoms might not have been captured. Finally, this study did not report symptom severity or impact on daily activities, thus the functional significance of these findings could not be assessed.

### Implications for Public Health Practice

Given the findings that approximately 16% of persons who have had an acute COVID-like illness might experience persistent symptoms through 12 months, post-COVID–like conditions could represent a substantial impact on health and the health care system. This report highlights the patterns of symptoms after acute COVID-like illness by providing estimates of symptom prevalence for both ongoing and emerging symptoms. Improved understanding of the persistent and fluctuating nature of symptoms could guide clinical care and public health response to post-COVID–like conditions.

## Footnotes

* The eight institutions are Rush University, Chicago, Illinois; Thomas Jefferson University, Philadelphia, Pennsylvania; University of California, Los Angeles, Los Angeles, California; University of California, San Francisco, San Francisco, California; University of Texas Southwestern Medical Center, Dallas, Texas; UTHealth Houston, Houston, Texas; University of Washington, Seattle, Washington; and Yale University, New Haven, Connecticut.

† Participants were eligible to enroll if they met the following inclusion criteria: 1) aged ≥18 years, 2) fluent in English or Spanish, 3) self-reported symptoms suggestive of acute SARS-CoV-2 infection at time of testing, and 4) received testing for SARS-CoV-2 with a Food and Drug Administration–approved or authorized molecular or antigen-based assay within the preceding 42 days. COVID test–positive and COVID test–negative participants were eligible to enroll. Participants with previous SARS-CoV-2 infection >42 days before enrollment and those without access to an internet-connected device for survey completion were excluded.

§ Participants were recruited through advertisements at testing sites, outreach through electronic health records (EHRs), and coordination with testing sites and health departments. To distinguish from asymptomatic persons being tested for exposure, surveillance, and preprocedural protocols, participants with acute COVID-like illness were enrolled by SARS-CoV-2 test result (positive or negative). Participants were asked to share access to their EHR data, which were used to verify SARS-CoV-2 infection status and to supplement vaccination data from surveys. If history of COVID-19 diagnosis or testing for SARS-CoV-2 information was unavailable in the EHR, participants were required to provide photographic proof of test results.

¶ Participants signed informed consent forms and completed a baseline survey and follow-up surveys every 3 months for up to 18 months postenrollment. All sites broadly recruited participants regardless of state of residence; there were no geographic or health system limitations.

**
https://www.cdc.gov/me-cfs/pdfs/wichita-data-access/symptom-inventory-doc.pdf

†† Symptom categories were any symptom (one or more symptoms), HEENT (headache, runny nose, loss of smell, loss of taste, sore throat, and loss of hair), constitutional (tired, chills, feeling hot, fever, and shakes), pulmonary (cough, shortness of breath, and wheezing), musculoskeletal (aches and joint pains), gastrointestinal (diarrhea, nausea or vomiting, and abdominal pain), cardiovascular (chest pain and palpitations), cognitive difficulties (forgetfulness/memory problems, difficulty thinking, or difficulty concentrating), and extreme fatigue (fatigue severity score ≥25).

§§ 45 C.F.R. part 46; 21 C.F.R. part 56.
